# A Challenging Case of Metastatic Crohn's Disease Without Gastrointestinal Manifestations

**DOI:** 10.7759/cureus.45791

**Published:** 2023-09-22

**Authors:** Ana S Pereira, Inês Coutinho

**Affiliations:** 1 Dermatology, Coimbra Hospital and University Center, Coimbra, PRT

**Keywords:** skin manifestations, cutaneous crohn’s disease, granulomatous inflammation, metastatic crohn’s disease, inflammatory bowel disease

## Abstract

Metastatic Crohn's disease (MCD) is a rare cutaneous manifestation of Crohn's Disease (CD), defined as non-caseating, granulomatous skin lesions non-contiguous with the gastrointestinal (GI) tract. Most patients with MCD either have concomitant classic manifestations of CD or develop them within a few months to years.

We report a case of MCD without known involvement of the GI tract, after more than three years from diagnosis. After failure or intolerance to several conventional treatments, including oral corticosteroids and azathioprine, adalimumab was initiated with a good response.

Diagnosis of cutaneous CD is made by a combination of clinical and histopathological findings. Therapeutic options include topical, intralesional, and systemic corticosteroids as well as topical and systemic immunosuppressants and immunomodulators. Surgical excision may be considered for refractory cases.

## Introduction

Crohn’s disease (CD) is defined as a chronic granulomatous inflammatory process affecting any segment of the gastrointestinal (GI) tract, which may present numerous extraintestinal manifestations, including to the skin. [1]

Dermatological manifestations of CD have a reported prevalence between 22 and 75% [1],^ ^and they may be generally subdivided into four subtypes: (1) lesions by direct extension from the bowel to the adjacent skin (the perianal or peristomal region), (2) non-contiguous skin lesions (known as metastatic CD), (3) non-specific or reactive dermatosis (such as erythema nodosum or pyoderma gangrenosum), and (4) secondary manifestations due to nutritional deficiencies or adverse reactions of the treatment [2].

Initially described by Parks et al. in 1965 [3], metastatic Crohn’s disease (MCD) is currently known as a rare entity with variable clinical manifestations and broad differential diagnosis [1]. Most patients with MCD have either previous or concomitant intestinal manifestations of CD, but some develop them months or years after the emergence of skin lesions, which makes the diagnosis particularly difficult [1]​​​​​​​. 

We present the rare case of MCD without known involvement of the GI tract at four-year follow-up.

## Case presentation

**Figure 1:**
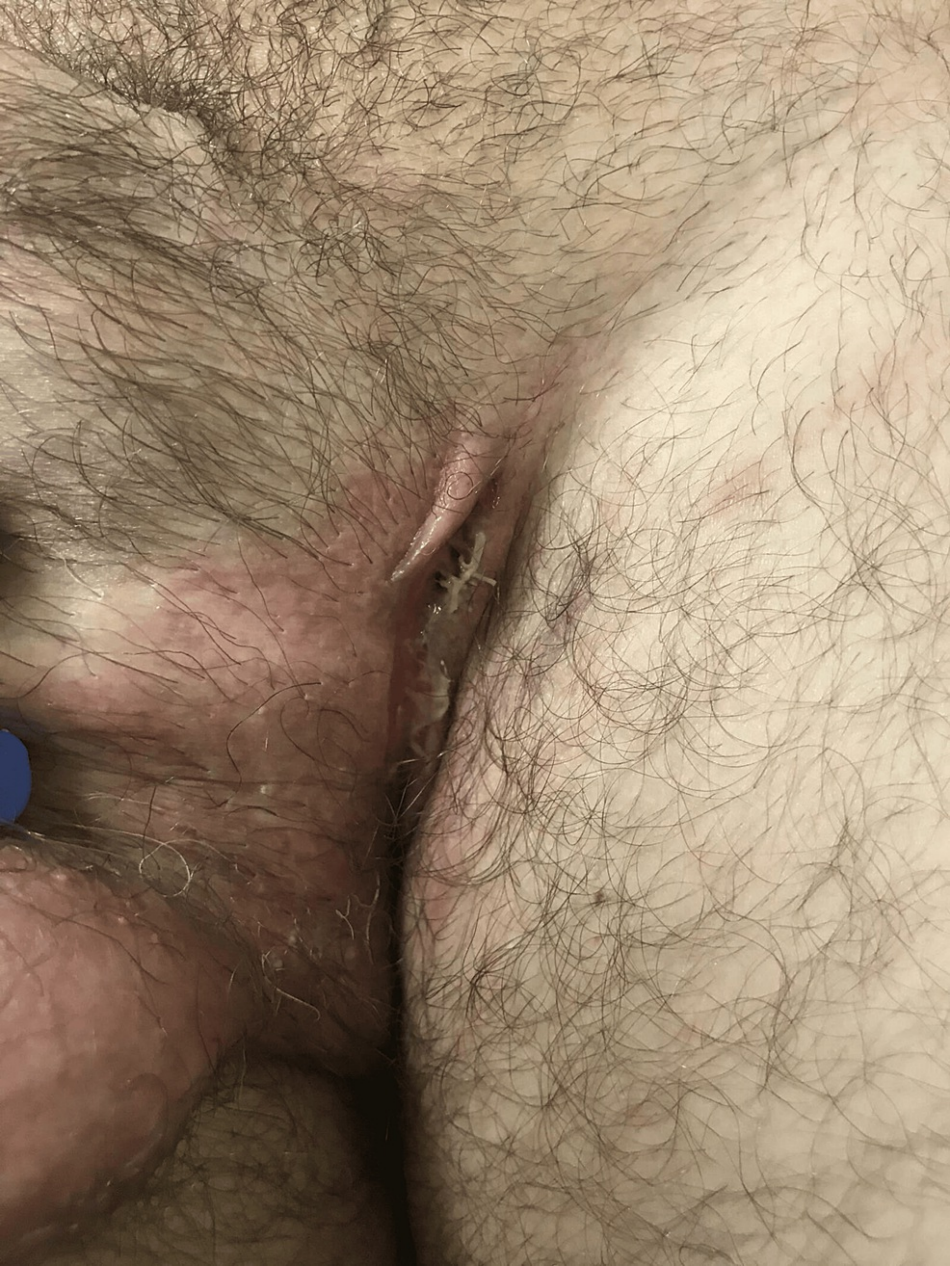
A linear fissure on the patient's left inguinal fold.

**Figure 2:**
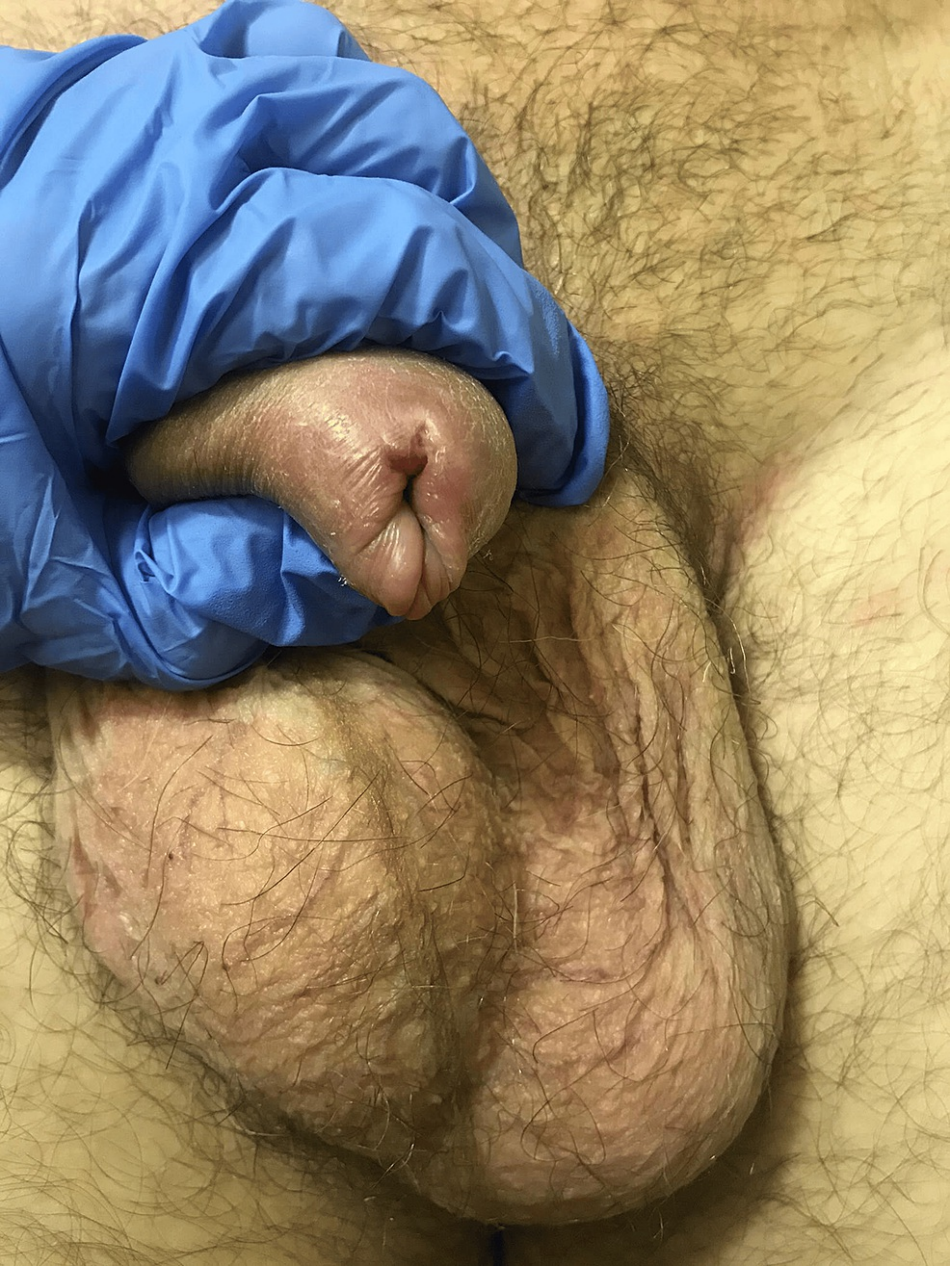
Penile edema.

**Figure 3:**
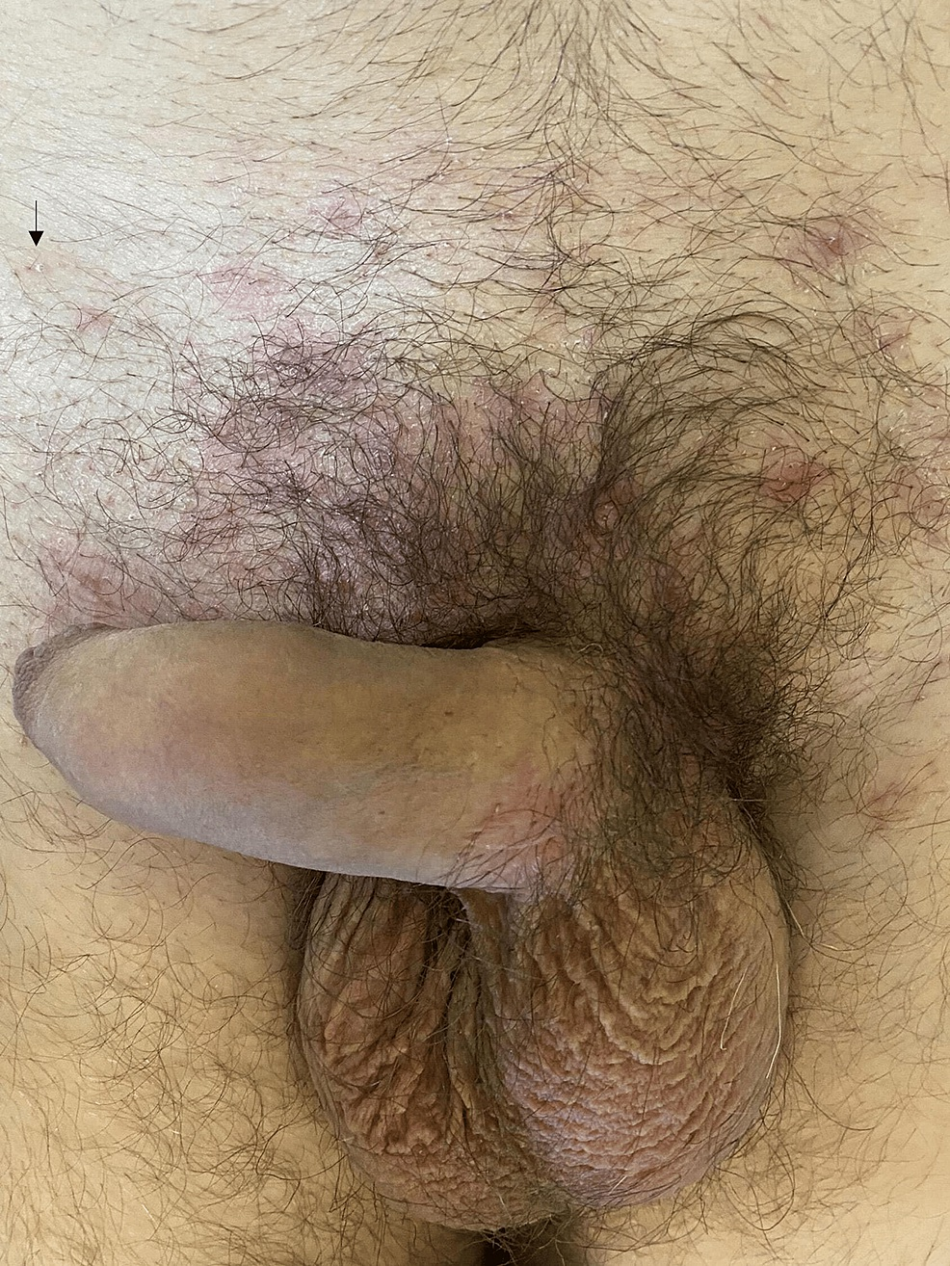
Numerous erythematous papules and pustules (arrow) on the pubic area.

A 36-year-old man presented to the Dermatology Department with a one-year history of knife-cut ulcers in both inguinal folds (Figure 1), marked edema of the penis (Figure 2), and papulopustular lesions scattered over the suprapubic area (Figure 3). On physical examination, no other mucocutaneous lesions were found and lymph nodes were not palpable.

The patient denied previous history of aphthous lesions or gastrointestinal complaints, including anal fistulas, pus, or pain. He also denied urinary symptoms (urethral discharge or dysuria), fever, or recent weight loss. His past medical and surgical history was unremarkable as was his family history. There was also no recent travel history or risky sexual behaviors.

The clinical setting had been previously interpreted as hidradenitis suppurativa and he had been medicated with several cycles of topical and oral antibiotics, with minor improvement and quick relapses.

Laboratory workup, including complete blood cell count with differential, routine chemistry of the blood and urine, erythrocyte sedimentation rate, and C-reactive protein, was normal. Rapid plasma reagin (RPR), viral markers (including HIV and hepatitis B and C), and the interferon-γ release assay for Mycobacterium tuberculosis were also negative.

**Figure 4:**
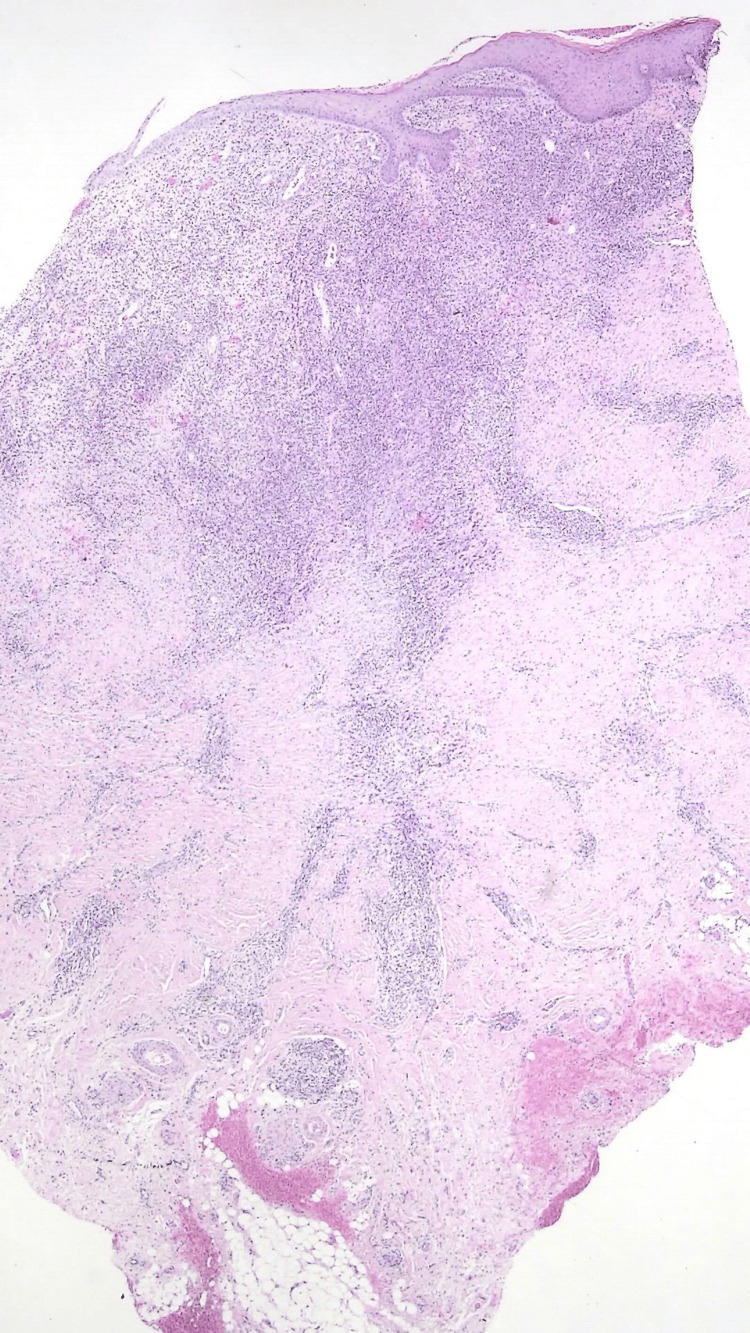
Irregular acanthosis with ulceration and a granulomatous infiltrate occupying the superficial and deep dermis (HE x10)

A skin biopsy from an inguinal lesion was performed and showed irregular acanthosis with an ulcerated epidermis, as well as a granulomatous infiltrate occupying the deep dermis, sometimes with perivascular arrangement (Figure 4).

**Figure 5:**
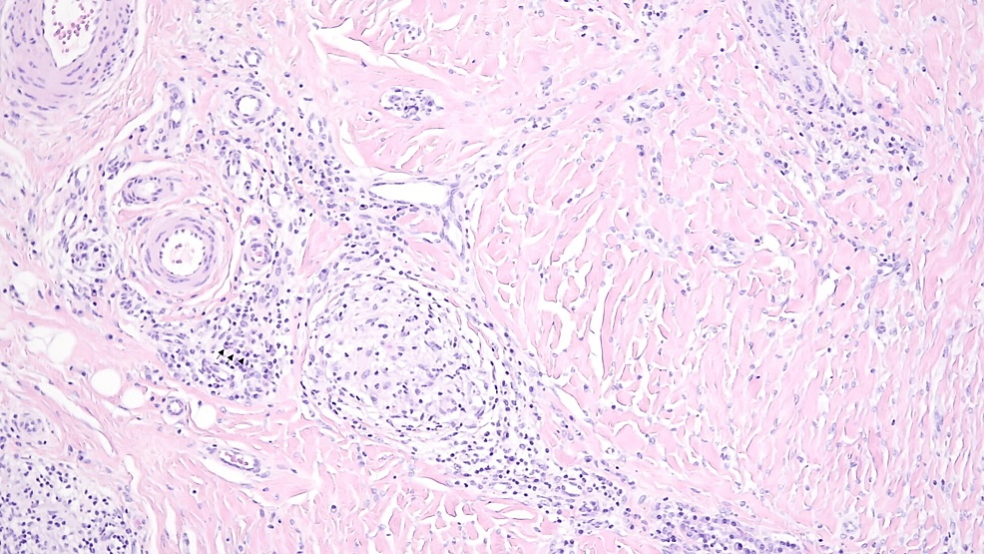
Granuloma formation on the deep dermis, surrounding the vessels, with multiple histiocytes and occasional plasma cells (arrows) (HE x40)

The well-circumscribed non-necrotizing granulomas were formed by histiocytes, multinucleated giant cells, lymphocytes, and occasional plasma cells (Figure 5).

Periodic Acid Schiff (PAS) and Ziehl-Neelsen stains did not reveal the presence of microorganisms and all skin cultures (mycobacterial, bacterial, and fungal) were negative.

After these results, the patient was evaluated by the gastroenterology department and underwent several gastrointestinal studies, including colonoscopy and CT enterography, which showed no alterations.

The correlation between clinical and pathological findings, in addition to the exclusion of all other possible causes, allowed the diagnosis of MCD despite a lack of evidence of gastrointestinal disease.

Given the extent of the lesions and their impact on the patient’s quality of life, he was medicated with oral methylprednisolone starting at 1mg/kg/day with gradual tapering and metronidazole 500 mg t.i.d. for four months, which provided a significant but transitory response.

Oral azathioprine was later tried at 100 mg/day but had to be suspended due to gastrointestinal intolerance.

Finally, biological therapy with adalimumab was initiated subcutaneously, 160 mg at week 0, 80 mg at week 2, and then 40mg every two weeks, with good and sustained improvement of the lesions to date.

The patient has been regularly monitored by both Dermatology and Gastroenterology departments, including yearly colonoscopies, but still without intestinal symptoms or lesions at four-year follow-up.

## Discussion

MCD is a rare dermatologic manifestation of CD [1], which may affect both sexes and all ages [4].

The pathogenesis of MCD remains uncertain [1,5]. It has been postulated that an unknown antigen from the GI tract deposited within the skin can trigger a T-helper cell type 1 (Th1)-mediated delayed hypersensitivity reaction responsible for the granuloma formation [1]. Depending on the proximity of the inflammatory reaction to a vessel, there may be vascular damage, a phenomenon known as granulomatous perivasculitis, which has been frequently found on histological examination of MCD lesions [4].

The clinical presentation of MCD is variable and depends on the affected site [1]. For instance, lesions affecting intertriginous areas or the genital skin are typically ulcerated due to maceration, while lesions on other locations frequently manifest as intact inflammatory papules and plaques [5].

According to a recent review [1], genital lesions prevail in both adults and children. These include ulceration, erythema, edema, and fissures on the vulva, labia, clitoris, scrotum, penis, or perineum, but also (less frequently) condyloma-like lesions, skin tags, or isolated lymphedema [5].

Extragenital MCD lesions are also highly polymorphous. They may present as erythematous to violaceous plaques and nodules, pustules, lichenoid papules, or even abscesses [5]. The legs are the main extragenital location affected by the disease, but lesions on the face, trunk, and breast have seldom been described [4].

The unspecific and variable clinical presentation of MCD leads to a wide differential diagnosis that may include hidradenitis suppurativa, intertrigo, pyoderma gangrenosum, and cellulitis, but also Behçet disease and sexually transmitted infections such as syphilis, lymphogranuloma venereum, and chronic herpes simplex [5].

The development of cutaneous lesions typically comes after the CD symptoms, which facilitates the diagnosis [1]. However, skin lesions may occur simultaneously or, more rarely, precede the onset of gastrointestinal manifestations [4]. In the latter case, the time span of subsequent onset of CD is 2 months to 4 years in adults as compared to 9 months to 14 years in children [6].

No correlation has been found between the activity of bowel disease and the occurrence of skin lesions [4], but it is known that patients with colonic involvement of CD are more likely to present MCD than patients with ileal disease alone [7].

On histology, MCD is defined by the presence of non-caseating sarcoidal granulomas on the superficial and deep dermis, with occasional extension into the subcutis [1].

The inflammatory infiltrate comprises mainly epithelioid and multinucleated histiocytes, as well as lymphocytes, plasma cells, and eosinophils, often surrounding dermal blood vessels (granulomatous perivasculitis) [5]. Neutrophils and foci of necrobiosis are rarely observed [5].

Other causes of granulomatous dermatitis, such as sarcoidosis, foreign-body granulomas, and mycobacterial and fungal infections, should be considered before assuming the diagnosis of MCD [7]. The distinction from cutaneous sarcoidosis is particularly challenging as it can be indistinguishable from MCD. Three specific features such as the presence of an ulcerated epidermis, an inflammatory infiltrate rich in eosinophils, and an evident dermal edema have been described as helpful in establishing the difference since they are usually absent in sarcoidosis [8].

Besides the lesional biopsy with special stains, a full diagnostic workup must include tissue cultures, serologic testing (Rapid plasma reagin/Treponema pallidum particle agglutination assay, hepatitis B and C, and human immunodeficiency virus) and interferon-γ release assay for Mycobacterium tuberculosis [5].

If these tests come back normal, MCD can be provisionally diagnosed and, for patients without previous history of CD, a gastroenterological evaluation including colonoscopy is mandatory [1,5].

MCD is mostly chronic and causes severe morbidity. Its treatment remains controversial given the absence of clinical trials, but a therapeutic algorithm for MCD has been proposed based on case series and case reports [5].​​​​​​​

Topical highly potent corticosteroids or intralesional corticosteroids are suggested as first-line options for localized disease, but oral metronidazole (800 to 1500 mg/d) may be beneficial as an “add-on” therapy [5]. On the other hand, in cases with multiple lesions, systemic corticosteroids may be considered first-line, using the lowest effective dose followed by a gradual taper to prevent relapses [5]. Other systemic drugs, such as azathioprine, cyclosporine, methotrexate, or thalidomide, have been used with variable responses [5].​​​​​​​

In more severe or refractory cases of MCD, tumor necrosis factor (TNF)-inhibitors, namely adalimumab and infliximab, have been used for decades with good efficacy and tolerability [5], but more recently other biological agents such as ustekinumab [9] and vedolizumab [10] have also been successfully used. Hyperbaric oxygen or surgical excision of skin lesions may be considered in severe or disfiguring cases when the medical treatment fails [2,5].

There are no established recommendations concerning the follow-up of patients with MCD without known intestinal disease, but multidisciplinary (dermatology and gastroenterology) long-term surveillance is essential.

## Conclusions

MCD is a rare event that even more rarely presents as the primary manifestation of CD.

Recognizing the clinical variability of MCD lesions and their main histological findings may be key to avoiding misdiagnosis and delayed treatment. The diagnosis of MCD should always be considered together with a wide list of differential diagnosis, all of which must be carefully excluded before assuming MCD, especially in cases without previous diagnosis of CD.

In addition to dermatological follow-up, these patients should be regularly monitored by a gastroenterologist since intestinal manifestations can appear months or years after skin lesions.
